# Alleviation of mycotoxin biodegradation agent on zearalenone and deoxynivalenol toxicosis in immature gilts

**DOI:** 10.1186/s40104-018-0255-z

**Published:** 2018-05-16

**Authors:** Donghui Shi, Jianchuan Zhou, Lihong Zhao, Xiaoping Rong, Yu Fan, Humera Hamid, Wenqiang Li, Cheng Ji, Qiugang Ma

**Affiliations:** 1 State Key Laboratory of Animal Nutrition, China Agricultural University, Beijing, 100193 People’s Republic of China; 2 Liaoning Medical University, Jinzhou, 121001 People’s Republic of China; 3 Fuqing Fengze Agricultural Science and Technology Development Co. Ltd., Fuzhou, 350011 People’s Republic of China; 4 State Key Laboratory of Direct-Fed Microbial Engineering, Beijing, 100193 People’s Republic of China

**Keywords:** Apoptosis, Deoxynivalenol, Mycotoxin biodegradation agent, Serum parameter, Vulva size, Zearalenone

## Abstract

**Background:**

The current study was carried out to evaluate the effects of mycotoxin biodegradation agent (MBA, composed of *Bacillus subtilis* ANSB01G and *Devosia* sp. ANSB714) on relieving zearalenone (ZEA) and deoxynivalenol (DON) toxicosis in immature gilts.

**Methods:**

A total of forty pre-pubertal female gilts (61.42 ± 1.18 kg) were randomly allocated to four diet treatments: CO (positive control); MO (negative control, ZEA 596.86 μg/kg feed and DON 796 μg/kg feed); COA (CO + 2 g MBA/kg feed); MOA (MO + 2 g MBA/kg feed). Each treatment contained 10 replicates with 1 gilt per replicate. Gilts were housed in an environmentally controlled room with the partially slatted floor.

**Results:**

During the entire experimental period of 28 d, average daily gain (ADG) and average daily feed intake (ADFI) of gilts in MO group was significantly reduced compared with those in CO group. The vulva size of gilts was significantly higher in MO group than CO group. In addition, significant increases in the plasma levels of IgA, IgG, IL-8, IL-10 and PRL were determined in MO group compared with that in CO group. ZEA and DON in the diet up-regulated apoptotic caspase-3 in ovaries and uteri, along with down-regulated the anti-apoptotic protein Bcl-2 in ovaries. The supplementation of MBA into diets co-contaminated with ZEA and DON significantly increased ADG, decreased the vulva sizes, reduced the levels of IgG, IL-8 and PRL in plasma, and regulated apoptosis in ovaries and uteri of gilts.

**Conclusions:**

The present results indicated that feeding diet contaminated with ZEA and DON simultaneously (596.86 μg/kg + 796 μg/kg) had detrimental effects on growth performance, plasma immune function and reproductive status of gilts. And MBA could reduce the negative impacts of these two toxins, believed as a promising feed additive for mitigating toxicosis of ZEA and DON at low levels in gilts.

## Background

Mycotoxins are toxic and secondary metabolites with low molecular weights generated by naturally occurring fungi belonging to the *Aspergillus*, *Penicillium* and *Fusarium* genera. Zearalenone (ZEA) is xenoestrogenic mycotoxin produced by several *Fusarium* species. The structure of ZEA is similar to that of 17β-oestradiol. ZEA can competitively bind to oestrogen receptors and activate the transcription of oestrogen-responsive genes [1]. Therefore, ZEA and some of its metabolites promote the development of hormone-dependent tumors [2]. Studies showed that ZEA oestrogenicity causes several functional and morphological changes in reproductive organs and leads to numerous reproductive problems in female rats and sows, such as induced lesions, ovarian dysfunction, false estrusembryotoxic effects, decreased fertility, early abortion [3–5]. Notwithstanding, there has been little studies to detect the apoptotic signal in the ovary or uterus of gilts exposed to ZEA in vivo. Deoxynivalenol (DON, vomitoxin) is a trichothecene mycotoxin commonly produced by *Fusarium* fungus as well. It is predominantly found in cereals and their byproducts. It has been reported that DON may cause a reduced feed intake, weight loss, growth retardation and impair function in vital organs such as liver and spleen [6, 7]. In addition, DON altered neuroendocrine signaling, proinflammatory gene induction, disruption of the growth hormone axis, and altered gut integrity [8–10].

The harmful effects of ZEA and DON on animal health have received a great attention worldwide. ZEA and DON occur widely in cereal food. Results from investigations showed that many feedstuffs for animals have been seriously contaminated globally with ZEA and DON [11]. It is revealed that human and animals are frequently exposed to low levels of mycotoxins due to the high rate of occurrence [12]. It is worth noting that intake of low levels of mycotoxins may also lead to damages of cells, tissues and organs, although they are too low to directly induce clinical symptoms on animals [13]. Besides, in many cases, the co-contamination of ZEA and DON is frequently observed, and exposing to both toxins simultaneously may exert synergistic or additive effects on animals. Therefore, it is very important to explore the influence of combined ZEA and DON at low levels on animals and find the appropriate detoxifying method in animal production. The co-contamination could inhibit oocyte maturation, reduce the antioxidant activities, decrease the efficacy of animal production, increase the health care and veterinary treatment costs, and decrease economic benefit [14]. Thus, minimizing the toxic effects of DON and ZEA in the contaminated cereals and feeds will be critical for animal performance and production.

Biodegradation is an efficient, specific and environmentally protective method to minimize the harmfulness of mycotoxins in both foods and feeds. Some microbes have been reported to possess various abilities regarding the degradation of ZEA, such as, *Rhodococcus pyridinivorans* [15], *Pseudomonas putida* [16] and *B. licheniformis* [17], as well as the degradation of DON, such as *Agrobacterium*–*Rhizobium* [18], *Nocardioides* [19] and *Eubacterium* [20]. Our research team have screened two bacterial strains (*B. subtilis* ANSB01G and *Devosia* sp. ANSB714) which could effectively degrade ZEA and DON respectively in vitro. *B. subtilis* ANSB01G could degrade 84.6%, 66.3% and 83.0% of the ZEA presenting in naturally contaminated corn, distiller’s dried grains with soluble (DDGS) and swine complete feed, respectively [21]. *Devosia* sp. ANSB714 originally isolated from the soil could degrade 97.3% DON within 24 h in the Lauria-Bertani medium [22]. We hypothesize that combination of *B. subtilis* ANSB01G and *Devosia* sp. ANSB714 (MBA) would be a potential tool for the detoxification of ZEA and DON at low levels in animal feeds and feedstuffs. Thus, the purpose of this experiment was evaluating the effects of the combination of *B. subtilis* ANSB01G and *Devosia* sp. ANSB714 on growth performance, reproductive physiological status, organ development such as vulva size, relative weights of genital organs, apoptosis in ovaries and uteri of immature gilts as well as the level of plasma immunology and oxidant stress in female gilts exposed to combined ZEA and DON for 28 d.

## Methods

### Experimental animals, diets and management

A total of forty immature gilts (Landrace × Yorkshire) with an average body weight of 61.42 ± 1.18 kg were randomly assigned to 4 dietary treatments with 10 gilts per treatment. The experiment consisted of an adaptation period of 3 d and an experiment period of 28 d. The experimental design followed a 2 (normal diet and moldy diet) × 2 (with and without supplementation of the biodegradation agent) factorial arrangement. The four treatment diets were: 1) a basal control diet contained 20% normal corn (CO); 2) a basal diet contained 20% moldy corn (MO); 3) the CO diet was supplemented with MBA (2 kg/t); and 4) the MO diet was supplemented with MBA (2 kg/t). All diets used in the study were formulated to be isocaloric and isonitrogenous included chlortetracycline in all diets. The basal diet met or exceeded NRC (2012) recommendations for all nutrients. The composition and nutrients of the basal diet were listed in Table 1.

**Table 1:** Ingredients and compositions of the basal diet

Ingredients	Percentage,%	Nutrition component	Content^1^
Wheat	36.50	DE, MJ/kg	13.10
Maize	20.00	Crude protein, %	17.65
Wheat middling	10.00	Lysine, %	1.02
Wheat bran	9.00	Methionine+Cystine, %	0.73
Dehulled soybean meal	10.00	Threonine, %	0.58
Rice bran	7.00	Tryptophan, %	0.20
Cottonseed meal	4.00	Calcium, %	0.74
Calcium hydrophosphate	1.00	Total phosphorus, %	0.71
Limestone	1.05	Non-phytatephosphorus, %	0.32
Salt	0.30	Sodium chloride, %	0.40
Threonine	0.05		
Lysine (70%)	0.60		
Vitamin Premix^2^	0.03		
Mineral Premix^3^	0.30		
Choline chloride	0.12		
Chlortetracycline	0.05		
Total	100.00		

^1^The value is calculated

^2^Provided per kg of diet: Vitamin A, 16,500 IU; Vitamin D_3_, 2,500 IU; Vitamin E, 18 IU; Vitamin K_3_, 2.3 mg; Vitamin B_1_, 3.1 mg; Pantothenic acid, 12 mg; Niacin, 27 mg; Folacin, 0.5 mg; Biotin, 0.1 mg; Vitamin B_12_, 0.02 mg; Vitamin B_2_, 5.5 mg; Vitamin B_6_, 3.3 mg

^3^Provided per kg of diet: Copper, 70 mg; Iron, 170 mg; Manganese, 30 mg; Zinc, 145 mg; Selenium, 0.41 mg; CoMBAlt, 0.07 mg; Iodine, 0.27 mg

Feed samples were taken at the beginning and end of the experiment for ZEA, aflatoxin (AF), DON, and ochratoxin A (OTA) analyses. The concentrations of ZEA and DON in the moldy corn were 2.65 and 4.01 mg/kg, respectively. The concentrations of ZEA + DON in CO, COA, MO and MOA diets were 90.68 μg/kg + 33 μg/kg, 89.88 μg/kg + 30 μg/kg, 596.86 μg/kg + 796 μg/kg and 600.99 μg/kg + 782 μg/kg, respectively. AF and OTA were not detected in any of the diets. The MBA was composed of 40% *B. subtilis* ANSB01G, 40% *Devosia* sp. ANSB714 and 20% carrier (rice husk meal) by industrial fermentation and dry-processing technologies. The *B. subtilis* ANSB01G was from one batch-fermented in a Luria-Bertani medium at 37 °C for 24 h and dried at 65 °C. The total viable counts of fermented-dried *B. subtilis* ANSB01G and fermented-dried *Devosia* sp*.* ANSB714 were both 1 × 10^9^ colony forming unit (CFU)/g.

Experimental procedures and swine care used in this study were in accordance with guidelines of the care and use of laboratory animals issued by the National Institute of Health [23] and by China’s Ministry of Agriculture. Gilts were penned (pen size, 0.55 m × 2.2 m) individually with ad libitum access to water. All gilts were fed individually diet of 3 kg daily provided in two equal portions (07:00 and 14:00 h). Body weights were measured at 0, 7, 14, 21 and 28 d. Feeds intakes and refusals were recorded daily with automatic feed system (Nedap Velos, Nedap China Ltd.). Average daily gains (ADG), average daily feed intake (ADFI) and feed: gain (F:G) were calculated.

### HPLC analysis

The concentration of ZEA, DON, AF and OTA in the diets were measured using HPLC method according to Chinese certification GB/T 23504–2009, GB/T 23503–2009, GB/T 18979–2003 and GB/T 23502–2009, respectively, with some small improvements [24]. The detection limits for these mycotoxins were 1.5 μg/kg for the ZEA, 0.1 μg/kg for the AF (AFB_1_, AFB_2_, AFG_1_, and AFG_2_), 0.02 mg/kg for the DON and 0.5 μg/kg for the OTA, respectively.

### Vulva size and organ weight determination

The length, width and height of vulva of pigs were measured at 0, 7, 14, 21 and 28 d for calculating the vulva volume as an approximately cylindroid shape (π × vulva length × vulva width × vulva height/4) according to the method described by Zhao et al. [24] with a slight modifications. Liver, heart, kidney, spleen and reproductive organs (ovary + cornua uteri + vagina) were examined macroscopically for an evaluation of their general appearances and then weighed separately. The weights were expressed on the basis of relative body weight (g/kg).

### Plasma immunology, antioxidant and hormone parameters

Samples of blood were obtained by venipuncture of the jugular vein after a 12-h fast at the end of the study. Blood was collected into vacutainers containing (Heparin sodium) as anticoagulant. Plasma was obtained by centrifugation at 3,000×*g* at 4 °C for 15 min and the immunology, antioxidant and hormone parameters were examined in the plasma samples.

The immunoglobulins including IgA, IgG and IgM were processed using the enzyme-linked immunosorbent assay (ELISA) kits (Nanjing Jiancheng Bioengineering Institute, Nanjing, China) following the manufacturer’s instructions and determined using a microplate reader (DNM-9202G, Beijing Perlang Co., Ltd., Beijing, China). The cytokines including ininterleukin-1β (IL-1β), interleukin-8 (IL-8) and interleukin-10 (IL-10) were measured using R-911 automatic radioimmunoassay counter (USTC Holdings Co., Ltd., China).

The activities of nitric oxide synthase (NOS), hydroxyl free radical (•OH), glutathione peroxidase (GSH-Px) and total superoxide dismutase (SOD) as well as the concentrations of malondialdehyde (MDA) in the plasma were measured using the commercial kits (Nanjing Jiancheng Bioengineering Institute, Nanjing, China) following the kit instructions.

The plasma estradiol (E2), luteotrophic hormone (LH), prolactin (PRL) and follicle-stimulating hormone (FSH) were measured via the commercial radioimmunoassay kits (Beijing Chemical in Biotech Co., Ltd., Beijing, China) following the manufacturer’s recommended procedure. Briefly, the samples were incubated with iodine (^125^I) in a monoclonal antibody solution. The incubated samples were then aspirated and the binding activity was measured using a gamma counter (γ-counter) (GC-1200, USTC Chuangxin Co., Ltd., Zonkia Branch, China). Hormone concentrations were measured according to each sample’s level of radioactivity.

### Protein extraction and western blotting in the ovary and uteri

The right parts of ovaries and uterus carefully dissected from 16 pigs (four pigs for each treatment) were snap frozen in liquid nitrogen and stored at − 80 °C until the analysis. The expressions of caspase-8, Bax, Bcl-2, and caspase-3 in the ovary and uteri were estimated using Western blotting assays. Total protein was extracted via a P1250 kit (Apply gen Technologies Inc., Beijing, China) and the equal amounts of the extracted protein (50 μg) were estimated using 15% SDS-polyacrylamide gel electrophoresis (SDS-PAGE) after centrifugation at 1,000×*g* at 4 °C for 15 min. The estimated protein then was transferred to the nitrocellulose membranes (Millipore Co., Billerica, MA, USA) at 80 V for 1 h. Membranes were blocked with blocking solution (5%, *w*/*v*, non-fat dried milk in the Phosphate Buffered Saline with Tween-20 (PBST)). Each membrane was incubated with a specific primary antibody caspase-8 (1:1,000 dilution), Bax (1:500 dilution), Bcl-2 (1:1,000 dilution) and caspase-3 (1:500 dilution) at 4 °C overnight. After three washes with PBST (15 min), the membrane was incubated with the diluted HRP-conjugated secondary antibody (diluted 1:2,500 in PBST) at room temperature for 1.5 h. The immunoblots were visualized by enhanced chemiluminescence reagent (ECL) following exposure of the filters to X-O mat Kodak autoradiography films. The bands were visualized and quantified using Image J 1.42 software. The signal intensity was normalized to β-actin (Boster Biological Technology Co., Ltd., Wuhan, China). All experiments were conducted in duplicate sets.

### Determination of zearalenone and its metabolites and deoxynivalenol in the liver

Zearalenone and its metabolites in the liver were analyzed using the method of Duca et al. [25] with a slight modification. Briefly, a quantity of 5.0 g of a grounded liver sample was mixed with 10 mL of a buffered solution of acetic acid-ammonium acetate (pH 4.8). This mixture was incubated for 15 h at 37 °C with 80 μL of a solution of β-glucuronidase/arylsulfatase with a pH 4.0 adjusted with glacial acetic acid. The mixture was then extracted with 10 mL of acetonitrile and 200 μL of NaOH solution (1 mol/L) while being stirred at 200 r/min for 60 min. The sample was centrifuged for 10 min at 5,000 r/min, and 10 mL of the supernatant was collected. The collected supernatant mixed with 40 mL of a phosphate-buffered saline solution (pH 7.4) and then filtered using glass fiber filter paper. The filtrate (40 mL) was passed through an immunoaffinity column (Clover Immuno Clean CF ZER, Clover, China) for the extraction and purification of ZEA and ZEA metabolites following the manufacturer’s instructions. The sample-loaded column was then washed with 10 mL of distilled water at 3 mL/min before the retained ZEA and ZEA metabolites were eluted using 2 mL of methanol. Then the methanol was evaporated to dryness under a gentle stream of nitrogen. The dried eluate was diluted in 200 μL of the mobile phase solution and 20 μL was injected into the HPLC system (Shimadzu LC-10 AT) for the separation and determination of the concentrations of ZEA and its metabolites.

Deoxynivalenol residues in the liver were analyzed using the method described by Zhao et al. [22]. Briefly, freeze-dried liver samples (0.5 g) were added with 60 μL of β-glucuronidase (EC 3.2.1.31, type H-2, 85,000 IU/mL, Sigma) and 3.0 mL of sodium acetate buffer (pH 5.5). The samples were extracted with a mixture of acetonitrile and water, followed by centrifugation at 5,000 r/min for 30 min after incubation at 37 °C for 12 h. After filtration, 2 mL of the extract was collected, with 8 mL of phosphate buffered saline (PBS, pH 7.4) containing 0.1% Tween-20 and centrifuged at 5,000 r/min for 30 min. The supernatant (8 mL) was passed through an immune-affinity column at a flow rate of 1 mL/min by gravity and washed with10 mL of PBS and 10 mL of double distilled water respectively, at a flow rate of 3 mL/min by gravity. DON was eluted using 2 mL of methanol, the eluent was evaporated by nitrogen flow stream at 40 °C, and the residue was dissolved in 100 μL of the mobile phase. Then, 20 μL of the eluent was injected into the HPLC system for the determination of the concentrations of DON.

### Statistical analysis

The data were analyzed as a 2 × 2 factorial design using the MIXED procedure of SAS 9.1 (SAS Inst., Inc., Cary, NC, USA); toxins level, MBA level, and their interaction were fixed factors, and experimental period and animal were random factors. When there was an interaction, post hoc Duncan’s multiple range tests were used to analyze differences among treatment means. A value of *P* < 0.05 was considered as statistically significant.

## Results and discussion

### Growing performance

Significant interactions of toxin and MBA were observed in ADG and ADFI of gilts in this study (*P* < 0.05, Table 2). The ADG and ADFI of gilts in MO group were lower than those in CO group (*P* < 0.05); however, the addition of MBA into moldy diets significantly improved the ADG (15.29%) (*P* < 0.05) and ADFI (6.22%) of gilts when compared with MO group, maintaining a similar status showed in CO group. The ADFI of gilts fed diet COA was decreased in comparison to the gilts fed diet CO (*P* < 0.05), but the ADG had no difference from those fed diet CO (*P* > 0.05). There was no significant interaction between mycotoxin and the biodegradation agent on F:G of gilts in this study (*P* > 0.05).

**Table 2:** Effects of MBA on production performance of immature gilts when exposed to ZEA and DON^1^

Diet treatments	Toxins, μg/kg feed		MBA, g/kg	Initial body weight, kg	ADG, kg	ADFI, kg	F:G
	ZEA	DON					
MO	596.86	796	0	62.26	0.85^b^	2.25^b^	2.67
MOA	600.99	782	2	60.89	0.98^a^	2.39^ab^	2.54
CO	90.68	33	0	62.32	0.95^a^	2.52^a^	2.65
COA	89.88	30	2	61.28	0.92^ab^	2.19^b^	2.39
Pooled SEM				2.70	0.03	0.07	0.08
Source of variation				*P*-value			
Main effect of toxins diets				0.93	0.24	0.64	0.30
Main effect of MBA level				0.66	0.32	0.18	0.02
Toxins diets × MBA level				0.95	0.04	< 0.01	0.42

^1^Each value is mean of ten replicates

*ZEA* zearalenone, *DON* deoxynivalenol, *MBA* mycotoxin biodegradation agent (*B. subtilis* ANSB01G + *Devosia* sp. ANSB714), *ADG* average daily gain, *ADFI* average daily feed intake, *F:G* feed:gain, *MO* the negative control diet, *MOA* the negative control diet plus 2 g MBA/kg diet, *CO* the positive control diet, *COA* the positive control diet plus 2 g MBA/kg diet, *pooled SEM* pooled standard error of the mean

^a-b^Means within a column with different letters differ significantly (*P* < 0.05)

The results clearly demonstrated that a diet contaminated with ZEA (596.86 μg/kg) and DON (796 μg/kg) had obviously negative effects on ADG and ADFI in the immature gilts. Similarly, Williams and Blaney [26] found that grower pigs fed a diet containing maize naturally contaminated with 11.5 mg/kg nivalenol (NIV) and 3 mg/kg ZEA had a deterioration of productive performance, such as decreased feed intake and ADG, in a 14-d experiment. The impact of ZEA + DON on eating behavior was also observed in our study and the ADFI of gilts fed with MO diet was decreased by 12% compared to the gilts fed with CO diet, indicating that combined DON and ZEA could induce anorexia of pigs. Clinical signs of hyperestrogenism and partial feed refusal were observed in gilts exposure to ZEA (64 mg/kg feed) and DON (4.5 mg/kg feed) [27]. It was reported that *B. subtilis* ANSB01G and *Devosia* sp. ANSB714 showed an effective ability to degrade ZEA and DON in vitro, respectively. Moreover, they displayed a high tolerance to simulated gastric fluid and intestinal fluid [21, 22]. In this study, *B. subtilis* ANSB01G and *Devosia* sp. ANSB714 could ameliorate the toxic effects of ZEA and DON in contaminated diet at low levels and improved the performance of gilts. It could be attributed to the ability of MBA to degrade ZEA and DON in the animal intestinal gut, consequently decreasing the toxicity of mycotoxin, which was consistent with the results of Zhao et al. [22, 24]*.* Different from previous study [24], chlortetracycline was added in all treatment diets in the current study; however, MBA could still alleviate the toxicosis caused by ZEA and DON, revealing that antibiotic would not influence the effectiveness of MBA.

### Vulva size and organ weight

The effects of MBA on the vulva size of immature gilts exposed to moldy diets containing ZEA and DON are presented in Table 3. The result indicated that vulva size increased progressively with the aging of the gilts. There was a significant interaction between mycotoxin and MBA on vulva size of gilts on 14, 21 and 28 d (*P* < 0.05). An obvious increase (*P* < 0.05) in the vulva sizes of the immature gilts was observed in MO group compared to those in CO group at 14, 21 and 28 d of the experiment. The vulva sizes observed in MOA group were markedly smaller than those in MO group (*P* < 0.05), but they were a little bit larger compared with those in CO group.

**Table 3:** Effects of MBA on vulva size of immature gilts when exposed to ZEA and DON (cm^3^)^1^

Diet treatments	Toxins, μg/kg feed		MBA, g/kg	Day of treatment				
	ZEA	DON		0	7	14	21	28
MO	596.86	796	0	3.53	6.10	7.75^a^	9.69^a^	9.75^a^
MOA	600.99	782	2	3.60	5.39	5.41^b^	6.65^b^	7.16^b^
CO	90.68	33	0	3.41	3.71	3.59^c^	4.23^c^	4.50^c^
COA	89.88	30	2	3.84	3.95	4.10^bc^	4.73^c^	4.26^c^
Pooled SEM				0.41	0.62	0.54	0.51	0.52
Source of variation				*P*-value				
Main effect of toxins diets				0.88	< 0.01	< 0.01	< 0.01	< 0.01
Main effect of MBA level				0.55	0.71	0.12	0.02	0.02
Toxins diets × MBA level				0.66	0.46	0.02	< 0.01	0.05

^1^Each value is mean of ten replicates

^a-c^Means within a column with different letters differ significantly (*P* < 0.05)

In vivo, 22.09 mg/kg ZEA in the diet caused negative alterations in the reproductive tract such as the uterus of pig, meanwhile this concentration of ZEA affected follicular and embryo development [28]. The clinical symptoms of ZEA toxicity in pigs consist of swelling of the vulva and prolapse of the vagina [24]. DON can also induce toxic effects on the ovaries, affecting follicular development and interfering with embryonic development in pigs [29, 30]. Vulva size increased linearly with the duration of feeding in gilts with diets containing 1.1 mg/kg of ZEA or greater [31]. In the present study, the vulva size measured on the day of 14, 21 and 28 were significantly smaller in the group MOA than that in MO group, supporting the fact that MBA can effectively mitigate the estrogenic swelling of the vulvas of immature female pigs caused by combined ZEA and DON.

No significant difference (*P* > 0.05) of relative organ weights were observed in liver, heart, kidney, spleen and reproductive organs (ovary + cornua uteri + vagina) among all groups (Table 4). Toxins in diets increased the relative organ weight of reproductive organs significantly (*P* < 0.05). And there was reduced tendency for the relative weight of reproductive organs in gilts from MOA group compared with MO group. The increase in liver weight was observed in growing bulls consumed a diet supplemented with *Fusarium* toxin-contaminated feed containing both ZEA (80–690 μg/kg DM) and DON (360–8,310 μg/kg DM) over a period of 10 wk [32]. Jiang et al. [31] reported that the relative weight of liver, kidney and genital organs of postweaning gilts increased linearly with the increase in dose of ZEA in the diet. The different results in different studies suggested that the animal species and age, the dose of toxin, and the source of toxin may influence the toxicity effect of ZEA and DON on organ weights.

**Table 4:** Effects of MBA on organ weight of immature gilts when exposed to ZEA and DON^1^

Diet treatments	Toxins, μg/kg feed		MBA, g/kg	Relative organ weight, g/kg				
	ZEA	DON		Liver	Heart	Kidney	Spleen	Reproductive organs
MO	596.86	796	0	19.46	1.77	3.39	3.48	2.40
MOA	600.99	782	2	18.94	2.00	3.27	3.99	1.84
CO	90.68	33	0	18.01	1.81	3.27	3.92	1.66
COA	89.88	30	2	18.45	1.69	3.43	3.24	1.51
Pooled SEM				0.71	0.12	0.13	0.23	0.16
Source of variation				*P*-value				
Main effect of toxins diets				0.36	0.43	0.92	0.65	0.04
Main effect of MBA level				0.97	0.75	0.93	0.80	0.16
Toxins diets × MBA level				0.65	0.33	0.48	0.10	0.40

^1^Each value is mean of ten replicates

### Plasma immunological parameters

Significant interactions between toxin and MBA were determined on the levels of plasma IgA, IgG, IL-8, and IL-10 (*P* < 0.05, Table 5). The levels of plasma IgA, IgG, IL-8 and IL-10 measured in gilts fed diet with low dose of ZEA + DON (596.86 μg/kg + 796 μg/kg) were greater than those in pigs fed positive control diet (*P* < 0.05). While the plasma levels of IgG, IL-8, IgA and IL-10 of gilts fed diet MOA were decreased by 11.22% (*P* < 0.05), 22.59% (*P* < 0.05), 8.52% and 8.59%, respectively, compared with those fed diet MO. There were no differences between the CO and COA groups in the levels of plasma IgA, IgM, IgG, IL-1β and IL-8 (*P* > 0.05).

**Table 5:** Effects of MBA on plasma immunological parameter of immature gilts when exposed to ZEA and DON^1^

Diet treatments	Toxins, μg/kg feed		MBA, g/kg	IgA, mg/g	IgM, mg/g	IgG, mg/g	IL-1β, pg/mg	IL-8, pg/mg	IL-10, pg/mg
	ZEA	DON							
MO	596.86	796	0	21.47^a^	21.60	255.12^a^	3.26	5.09^a^	3.84^a^
MOA	600.99	782	2	19.64^ab^	21.78	226.50^b^	2.63	3.94^b^	3.51^ab^
CO	90.68	33	0	17.25^b^	19.65	195.44^c^	1.73	2.84^c^	2.19^c^
COA	89.88	30	2	19.55^ab^	17.76	197.63^c^	1.71	2.99^c^	3.03^b^
Pooled SEM				0.72	1.82	6.48	0.15	0.09	0.21
Source of variation				*P*-value					
Main effect of toxins diets				0.02	0.14	< 0.01	< 0.01	< 0.01	< 0.01
Main effect of MBA level				0.75	0.65	0.08	0.06	< 0.01	0.27
Toxins diets × MBA level				0.02	0.59	0.04	0.08	< 0.01	0.02

^1^Each sample was assayed in triplicate

^a-c^Means within a column with different letters differ significantly (*P* < 0.05)

Our results demonstrated that plasma immunoglobulin concentrations were significantly influenced by dietary ZEA and DON. Similar results were also noticed in previous reports, in which the level of the plasma IgA was increased in rats due to ZEA exposure [33, 34]. The reason for this outcome was presumably that the increasing cytokines production promoted the generation of IgA and IgG in plasma of pigs exposed to the combined ZEA and DON. Cytokines exert an important function in the regulation of the immune response. In this study, toxins in diets caused obvious elevation of both pro- and anti-inflammatory cytokines (IL-1β, IL-8 and IL-10) in plasma of gilts. Any of cytokines IL-10 and IL-1β could directly or indirectly enhance differentiation of IgA-secreting B cells. It was reported that plasma IL-10 and IL-1β were increased when exposed to ZEA [33, 35]. The expression and synthesis of IL-1β and IL-8 were increased when exposure to ZEA-contaminated feed [34, 36]. Our observation gains strong support from these above reports. In this trial, MBA could reduce the elevated levels of IgG and IL-8, thus protecting the immune system of gilts from damage caused by combined ZEA and DON.

### Plasma oxidant stress parameters

No significant interactions were identified between toxin and MBA on the plasma antioxidant indices (Table 6). However, the highest levels of plasma NOS, •OH and MDA, along with the lowest levels of plasma GSH-Px and SOD were observed in group MO.

**Table 6:** Effects of MBA on serum antioxidant index of immature gilts when exposed to ZEA and DON^1^

Diet treatments	Toxins, μg/kg feed		MBA, g/kg	NOS, IU/mg	•OH, IU/mg	GSH-Px, IU/mg	SOD, IU/mg	MDA, μmol/mg
	ZEA	DON						
MO	596.86	796	0	2.25	25.78	75.73	4.44	924.99
MOA	600.99	782	2	2.09	23.04	81.29	5.32	815.11
CO	90.68	33	0	1.79	17.80	87.92	5.83	645.99
COA	89.88	30	2	1.69	17.64	100.85	6.18	618.15
Pooled SEM				0.29	0.97	2.21	0.32	19.26
Source of variation				*P*-value				
Main effect of toxins diets				0.18	< 0.01	< 0.01	< 0.01	< 0.01
Main effect of MBA level				0.66	0.17	< 0.01	0.09	< 0.01
Toxins diets × MBA level				0.92	0.22	0.13	0.43	0.07

^1^Each sample was assayed in triplicate

Oxidant stress can cause damage to all components in cells including cleavage of proteins, endogenous DNA lesions and lipid peroxidation. Reactive oxygen species (ROS) such as hydroxyl radical (•OH) and hydrogen peroxide (H_2_O_2_) are the primary products generated from these damages. A great attention has been paid to the oxidative stress induced by *Fusaria* mycotoxins in the last decades. For example, dietary ZEA induced serious oxidative injuries in both renal and hepatic tissues in mice via adverse impact upon the level of the MDA, and the activities of CAT and SOD [37]. GSH-Px and SOD are the key antioxidant enzymes that can scavenge ROS generated from oxidant stress. Consequently, the activities of GSH-Px and SOD have been recognized as the leading parameters of anti-oxidative stress. Ren et al. [38] reported that the concentration of ZEA + DON (2.5 mg/kg BW and 30 mg/kg BW) showed more obvious effects on the dysregulation of splenic antioxidant functions, like as •OH inhibition capacities. However, there was no significant difference in the plasma antioxidant indices among treatment groups in our research, which probably related to the low dose of ZEA and DON.

### Plasma hormone parameters

The present outcomes implied that immature gilts exposed to ZEA and DON (596.86 μg/kg + 796 μg/kg) exhibited a noticeably increased concentration of plasma PRL (*P* < 0.05, Table 7). But the level of serum PRL in MOA group supplemented with MBA was significantly decreased than that in MO group without MBA (*P* < 0.05). No significant difference was observed in levels of plasma FSH, LH, and E2 among all the groups (*P* > 0.05). No differences were observed in the levels of E2, LH, PRL and FSH between the group CO and COA (*P* > 0.05), either.

**Table 7:** Effects of MBA on plasma hormone of immature gilts when exposed to ZEA and DON^1^

Diet treatments	Toxins, μg/kg feed		MBA, g/kg	E2, pg/mL	LH, mIU/mL	PRL, µIU/mL	FSH, mIU/mL
	ZEA	DON					
MO	596.86	796	0	25.87	23.04	166.94^a^	17.04
MOA	600.99	782	2	29.29	23.74	149.22^b^	18.24
CO	90.68	33	0	30.65	24.96	118.35^c^	18.20
COA	89.88	30	2	31.35	25.80	121.83^c^	17.89
Pooled SEM				0.71	1.16	3.79	1.14
Source of variation				*P*-value			
Main effect of toxins diets				< 0.01	0.13	< 0.01	0.73
Main effect of MBA level				0.02	0.53	0.10	0.71
Toxins diets × MBA level				0.09	0.95	0.02	0.53

^1^Each sample was assayed in triplicate

^a-c^Means within a column with different letters differ significantly (*P* < 0.05)

Numerous researches demonstrated that ZEA binds to estrogen receptors, generating an estrogen-like response and causing serious hyperestrogenism in several animal species, particularly in pigs [39]. The immature gilts appeared to be more predisposed to ZEA insult during immature than other growth stage. This is in agreement with Chen et al. [40] who observed that serum levels of prolactin in gilts fed a diet with 2.0 mg/kg ZEA were significantly increased. Another study also reported a significant increase in the serum level of PRL in pigs consuming a diet containing 238.57 μg/kg of ZEA in comparison to diet without ZEA [24].

### Apoptotic examination

**Fig. 1:**
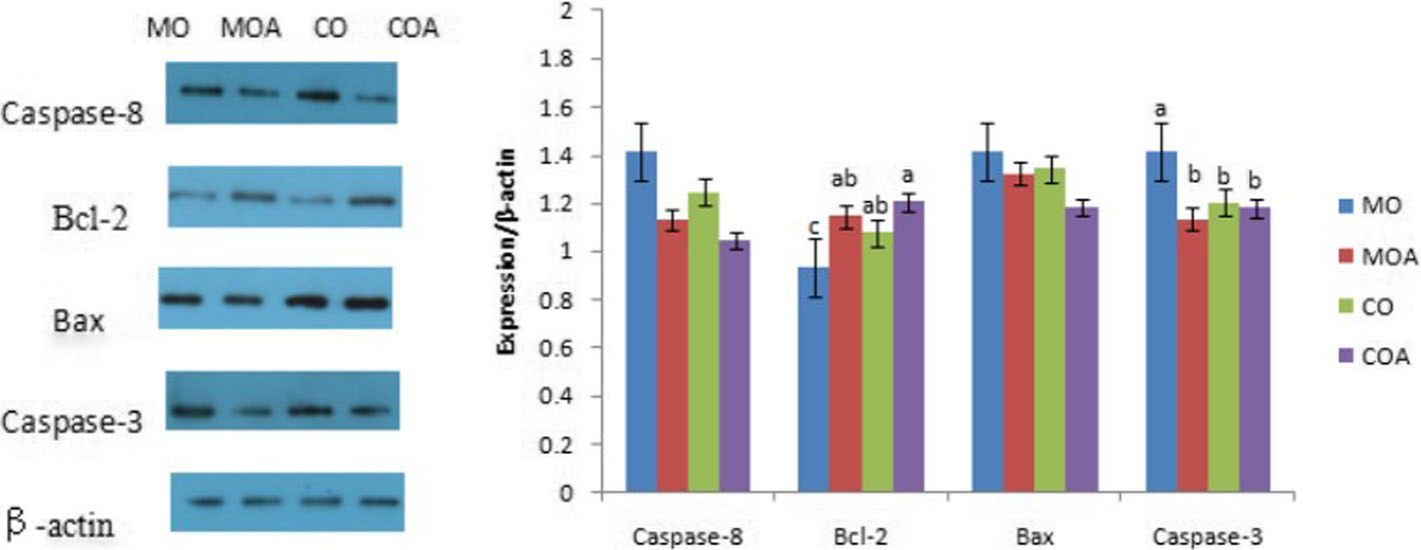
Effects of MBA on expressions of apoptosis-regulatory proteins in ovaries of immature gilts when exposed to ZEA and DON. MO = the negative control diet; MOA = the negative control diet plus 2 g MBA/kg diet; CO = the positive control diet; COA = the positive control diet plus 2 g MBA/kg diet. ^a-c^Columns with different letters differ significantly (*P* < 0.05)

**Fig. 2:**
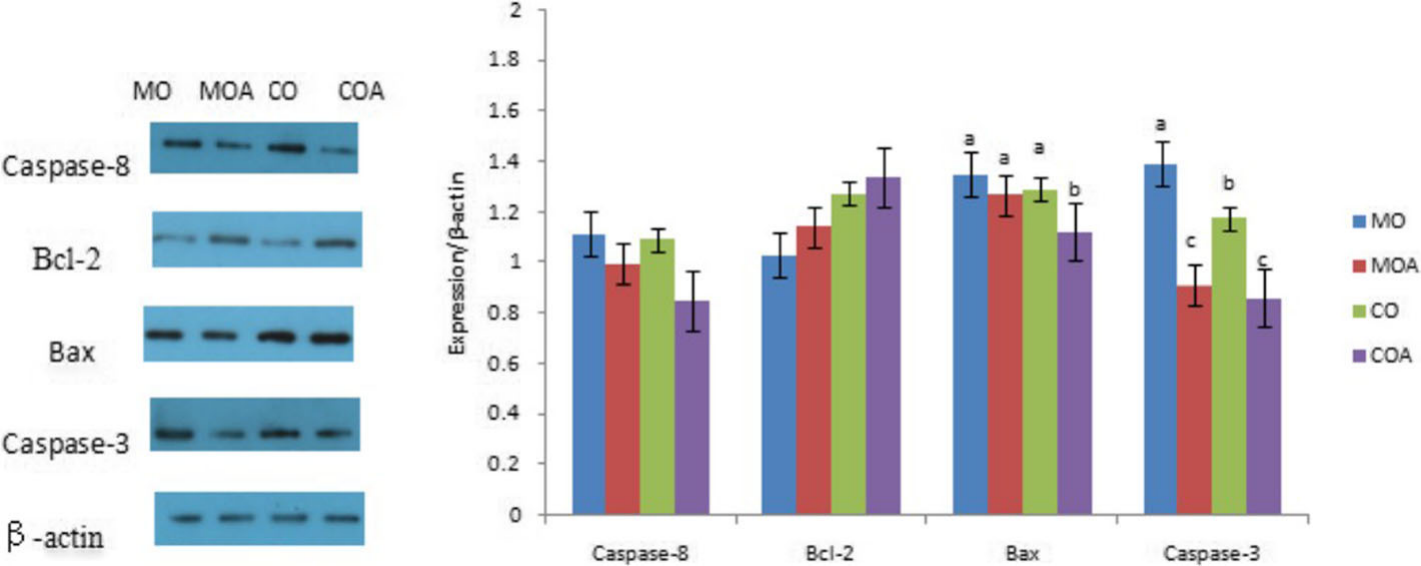
Effects of MBA on expressions of apoptosis-regulatory proteins in uterus of immature gilts when exposed to ZEA and DON. MO = the negative control diet; MOA = the negative control diet plus 2 g MBA/kg diet; CO = the positive control diet; COA = the positive control diet plus 2 g MBA/kg diet. ^a-c^Columns with different letters differ significantly (*P* < 0.05)

The results of western blotting assay for caspase-8, Bcl-2, Bax and caspase-3 proteins in ovaries of gilts are depicted in Fig. 1. The expressions of caspase-3 protein in ovaries of gilts fed MO diet were higher, while the expressions of Bcl-2 protein were lower than those fed CO diet (*P* < 0.05). The incorporation of MBA into MO diet had alleviated the negative effects of ZEA and DON on these protein expressions in ovaries of immature gilts. There were similar effects of mycotoxin and MBA on the expression of these proteins in the uterus of pigs, displayed in Fig. 2.

The initiator caspase-8 can directly activate pro-caspase-3 without any accelerator [41]. Caspase-3 is considered to be the key protein in the execution of apoptosis [42], as well as both pro-apoptotic (Bax) and anti-apoptosis (Bcl-2) are decisive factor and play a pivotal role in determining whether cells survival or death. Bcl-2 can inhibit cytochrome C translocation, thereby block caspase activation and the apoptotic process [43]. In the present study, ZEA and DON have up-regulated the expression of apoptotic protein caspase-3 in ovaries and uteri, as well as down-regulated the expression of anti-apoptotic protein Bcl-2 in ovaries of gilts. DON can significantly increase the protein levels of p53 and Bax/Bcl-2 ratio in mouse thymic epithelial cell line 1 and induce mitochondrial dysfunction [44]. ZEA can induce apoptosis of porcine granulosa cells in a dose-dependent manner by a caspase-3- and caspase-9-dependent mitochondrial pathway [45]. The result of our study is reinforced by the reports above. In the present analysis, the up-regulated protein caspase-3 and down-regulated protein Bcl-2 may accelerate the apoptotic process in ovaries and uteri of gilts. It is determined that apoptosis in the ovary and the uterus is linked to hormonal secretion, which was potentially related to ZEA in diets which binds to oestrogen receptors and activates the transcription of oestrogen-responsive genes, thereby affects endocrine. Furthermore, there was a significant mutual interaction between toxin and MBA on Bcl-2 in ovaries and caspase-3 in uteri of immature gilts. It indicated that adding MBA in moldy diet contaminated with low levels of ZEA and DON may be an effective approach to recover the expression levels of these apoptosis-related proteins in ovaries and uteri of gilts.

### Determination of ZEA and its metabolites and DON in the liver

Neither ZEA and its metabolites (α-zearalanol (α-ZAL), β-zearalanol (β-ZAL), α-zearalenol (α-ZOL), β-zearalenol (β-ZOL)) nor DON were detected in liver samples collected in this study, in agreement with the result of Zhao et al. [24]. It was difficult to find the metabolites of these two kinds of mycotoxin in the liver, serum or muscle tissues of the animal due to the low concentration (less than 1 mg/kg) in the diet.

## Conclusion

Obvious interactions between toxin and MBA on ADG, ADFI, vulva size, the levels of IL-8 and serum PRL, Bcl-2 in ovaries, and caspase-3 in ovaries and uteri in the immature gilts are found in our study. These significant connections indicated that combined ZEA and DON at low levels in diet had negative effectiveness on the above index, whereas the addition of MBA (*Bacillus subtilis* ANSB01G and *Devosia* sp. ANSB714 mixed in ratio) ameliorated these damages and cytotoxicity in ovaries and uteri induced by dietary toxins. Therefore, MBA used in this study was suggested as a potential application for detoxification of ZEA and DON at relatively low levels in animal production.

## Abbreviations

ADFI: Average daily feed intake
ADG: Average daily gain
AF: Aflatoxin
BW: Body weight
CFU: Colony forming unit
DDGS: Distiller’s dried grains with soluble
DM: Dry matter
DON: Deoxynivalenol
E2: Estradiol
ECL: Enhanced chemiluminescence reagent
ELISA: Enzyme-linked immunosorbent assay
F:G: Feed:gain
FSH: Follicle-stimulating hormone
GSH-Px: Glutathione peroxidase
IL-10: Interleukin-10
IL-1β: Ininterleukin-1β
IL-8: Interleukin-8
LH: Luteotrophichormone
MBA: Mycotoxin biodegradation agent
MDA: Malondialdehyde
NOS: Nitric oxide synthase
OH: Hydroxyl free radical
OTA: Ochratoxin A
PBST: Phosphate buffered saline with Tween-20
PRL: Prolactin
SDS-PAGE: SDS-polyacrylamide gel electrophoresis
SOD: Superoxide dismutase
ZEA: Zearalenone
